# Negative pressure wound therapy for flap closed-incisions after 3D-printed prosthesis implantation in patients with chronic osteomyelitis with soft tissue defects

**DOI:** 10.1186/s12891-023-06970-1

**Published:** 2023-10-19

**Authors:** Zhiyu Lin, Xinling Zhang, Yujie Chen, Yun Tian, Xin Yang, Zhenmin Zhao

**Affiliations:** 1 Department of Plastic Surgery , Peking University Third Hospital, No. 49 North Garden Road, Haidian District, Beijing, 100191 China; 2 Department of Plastic Surgery, Beijing Hospital, Beijing, China; 3 Department of Orthopaedics, Peking University Third Hospital, No. 49 North Garden Road, Haidian District, Beijing, 100191 China

**Keywords:** Chronic osteomyelitis, Closed-incision, Negative pressure wound therapy, Soft tissue defects

## Abstract

**Background:**

The flap closed-incisions healing after 3D-printed prosthesis implantation in Chronic Osteomyelitis with Soft Tissue Defects (COSTD) is critical. This study aimed to explore the safety and effectiveness of Negative Pressure Wound Therapy (NPWT) in promoting flap closed-incisions healing.

**Methods:**

Retrospective analysis of clinical data was performed, including baseline, surgical and hospitalization information. The efficacy of NPWT was assessed by comparing the ASEPSIS scores, Visual Analogue Scale (VAS), Activity of Daily Living Scale (ADLS), and Lower Extremity Functional Scale (LEFS), as well as the major postoperative complications.

**Results:**

The study included 20 patients, 13 received conventional dressing (Control group) and 7 received NPWT treatment (NPWT group). These two groups exhibited a notable disparity in the distribution of ASEPSIS scores, and the median scores were 24 in Control group and 9 in NPWT group (*p* = 0.001). Eight patients in the Control group experienced major incisional complications, including 7 cases of exudation, 3 cases of infection, 2 cases of non-healing, and 1 case of dehiscence, while none were observed in the NPWT group (*p* = 0.015). The VAS, ADLS, and LEFS scores were significantly improved in the NPWT group compared to the Control group (*p* = 0.003, 0.017, and 0.043, respectively).

**Conclusions:**

The study findings suggest that NPWT applied to the healing process of flap closed-incisions after 3D prosthesis implantation in patients with COSTD can reduce the occurrence of postoperative major complications and promote the recovery of lower limb function and daily activities, which should be recommended for clinical practice.

## Background

The incidence of chronic osteomyelitis in the lower extremities of adults is rising every year with the increase of traffic-related trauma, medical prosthetic infections and peripheral vascular insufficiency [1]. Chronic osteomyelitis is an inflammatory process caused by pathogenic bacterial infections, accompanied by bone and even surrounding soft tissue defects [2, 3]. Its treatment involves the systematic use of antibiotics in combination with surgical debridement to remove necrotic bone and infected soft tissue [4]. Our center has established a sequential treatment process for Chronic Osteomyelitis with Soft Tissue Defects (COSTD). The process includes the use of antibiotics, bone cement spacer implantation, complete debridement of the infected site, flap reconstruction of the soft tissue wound, and finally implantation of 3D-printed prostheses [5]. The full course of treatment is characterized by long duration, high difficulty, and expensive cost, causing huge economic burdens to health care system as well as significant decreased patient quality of life.

The satisfactory healing of flaps after the implantation of 3D-printed prostheses plays a crucial role in sequential treatment. Failure to achieve proper healing not only causes secondary damage to the flaps but also alters the surrounding environment of the flaps, potentially affecting blood supply and leading to postoperative complications such as flap breakdown, exudation, poor healing, infection, incomplete coverage of the prostheses, or even necrosis [6]. These complications can result in the failure of early-stage treatments, causing significant time and economic costs, as well as serious loss of shape and function. Therefore, it is an urgent clinical problem to improve the healing rate of secondary flap closed-incisions and reduce the incidence of flap-related complications in the therapy of COSTD.

Negative pressure wound therapy (NPWT) has been shown in previous studies to promote wound healing by enhancing the inflammatory response, accelerating granulation growth, and boosting angiogenesis [7]. However, it remains unclear whether NPWT can promote flap closed-incisions healing and reduce complications after the implantation of 3D-printed prostheses. Thus, this study aimed to investigate the safety and efficacy of NPWT during the healing of flap closed-incisions after the implantation of 3D-printed prostheses by reviewing medical records of patients with COSTD.

## Materials and methods

### Patient

This study complied with the ethical rules for human experimentation that are stated in the 1975 Declaration of Helsinki and was approved by the Ethics Committee of Peking University Third Hospital (Ethics No. IRB00006761-M2020576). We retrospectively analyzed 20 patients with COSTD who underwent sequential treatment at our center, 7 used NPWT to cover the flap closed-incisions after implantation of 3D-printed protheses and 13 used conventional dressing. The informed consents were obtained from all of the patients.

Inclusion criteria: (a) age ≥ 18 years; (b) a clear diagnosis of chronic osteomyelitis with bone defect and soft tissue defect in lower limbs; and (c) the wound healed well after bone cement Spacer placement accompanied by the flap covering for at least half a year.

Exclusion criteria: (a) local soft tissue wounds or defects caused by other reasons, such as skin and soft tissue malignant tumors; (b) bone defect caused by other reasons, such as bone tumor, bone tuberculosis; (c) delayed implantation of 3D-printed protheses over six months for various reasons; and (d) follow-up data were missing.

### Collection of clinical data

Patients’ data including basic information, operation time, intraoperative bleeding, laboratory indices, hospitalization time and cost, functional scores and satisfaction rating were collected using the hospital electronic medical record system and outpatient follow-up. The follow-up period was from the immediate implantation of 3D-printed to six months after the surgery.

The major flap-related complications, including exudation, hematoma, infection, dehiscence, and non-healing, were observed and recorded during the follow-up period. The non-healing was defined as the wound was not fully healed until one month after suture. The ASEPSIS score is utilized to assess postoperative wound complications and infection status. ASEPSIS was initially proposed by Wilson et al. and is employed in the evaluation of surgical site infections [8]. The scoring is performed for 5 out of the first 7 postoperative days. The methodology involves assigning a score ranging from 0 to 5 for erythema and serous exudate, and a score ranging from 0 to 10 for purulent exudate and deep tissue separation, depending on the percentage of the affected wound. Additional penalty points are added to the 5-day score to obtain an overall ASEPSIS score. These penalty points are allocated for antibiotic treatment of wound infection (10), drainage of pus under local anesthesia (5), debridement of wound under general anesthesia (10), isolation of bacteria from the wound (10), and hospitalization exceeding 14 days (5). Subsequently, wounds are classified based on the total ASEPSIS score: 0–10, satisfactory healing; 11–20, disturbance of healing; 21–30, minor wound infection; 31–40, moderate wound infection; and greater than 40, severe wound infection (Table 1).

**Table 1:** ASEPSIS scoring system

Wound characteristic	Proportion of wound affected (%)					
	0	<20	20–39	40–50	60–70	>80
Serous exudate	0	1	2	3	4	5
Erythema	0	1	2	3	4	5
Purulent exudate	0	2	4	6	8	10
Separation of deep tissues	0	2	4	6	8	10
Extra penalty points						
Scoring criteria					Points	
Antibiotics					10	
Drainage of pus under local anaesthetic					5	
Debridement of wound under general anaesthetic					10	
Serous discharge					Daily 0–5	
Erythema					Daily 0–5	
Purulent exudate					Daily 0–10	
Separation of deep tissues					Daily 0–10	
Isolation of bacteria from discharge					10	
In-patient stay in excess of 14 days					5	

### Efficacy evaluation

The efficacy evaluations were conducted preoperatively and at 3 months postoperatively, respectively. The subjective satisfaction of patients before and after implantation of the flap was collected using the Visual Analogue Scale (VAS), with 0 being very dissatisfied and 10 being very satisfied. The Activity of Daily Living Scale (ADLS) was used to assess the patients' daily living status with a full score of 100. The Lower Extremity Functional Scale (LEFS) was used to evaluate the postoperative function of the patients, with a full score of 80. And all the change in VAS (ΔVAS), ADLS (ΔADLS) and LEFS(ΔLEFS) were calculated. The overall flap blood supply was reflected by four factors: postoperative flap swelling, epidermal blisters, skin edge color and skin temperature.

### Surgical treatment

**Fig. 1:**
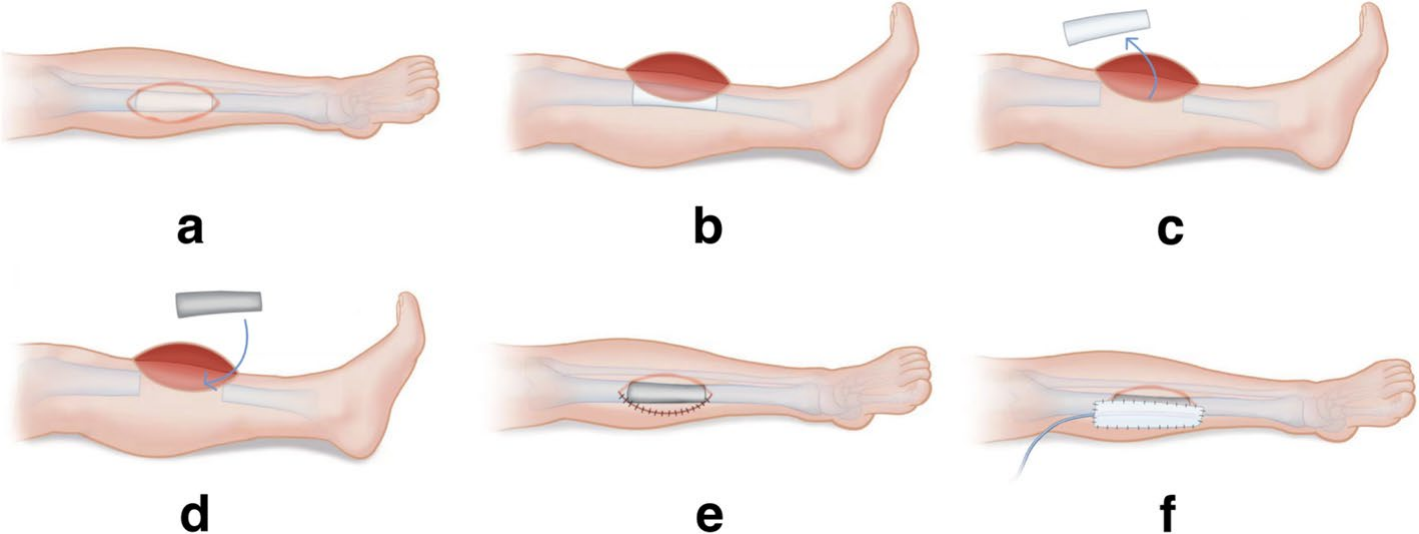
The schematic of surgical procedure. **a** Following at least six months of appropriate healing after bone cement Spacer placement, the surgical incision is designed to coincide with the scar from the previous flap incision. **b**-**c** The flap edge is incised, and the bone cement Spacer is fully extracted. **d** A 3D-printed prosthesis is inserted. **e** The flap incision is closed with interrupted sutures, and (**f**) a negative-pressure wound therapy (NPWT) is applied over the flap closed-incision

Patients were divided into NPWT group and Control group. The preliminary surgical operations were the same in both groups: (1) implant the bone cement Spacer and turn the flap to cover the wound; (2) design the location and size of the incision at the flap edge according to the size of the 3D-printed prosthesis at least six months after the wound heals well; (3) remove the bone cement Spacer and implant the 3D-printed prosthesis; (4) Rinse the wound, stop bleeding sufficiently, place drainage next to the prosthesis, and suture the skin incision after interrupted suturing of the subcutaneous tissue (Fig. 1). After completing the above operations, the Control group used gauze to cover the wound surface and subsequently took conventional dressing, while the NPWT group used VSD device (Wuhan Visdi Medical Technology Co., Ltd.) to cover the flap closed-incisions, so that the tissue at the flap incision was evenly compressed to avoid local pressure and jamming. Continuous negative pressure drainage was applied with 33.2 to 39.8 kPa. The VSD devices were removed after 2 weeks.

### Data analysis

Data were analyzed using Statistical Product and Service Solutions software (SPSS, version R26.0.0.2, IBM). Continuous variables are expressed as the mean ± standard deviation, and classified variables are expressed as frequencies and/or percentages. For intergroup or intragroup parameter comparisons, independent or paired sample t tests were performed for data subject to a normal distribution, and independent or paired sample Mann‒Whitney U tests were performed for data not subject to a normal distribution. Count data were tested by the χ^2^ test or Fisher’s test. *P* < 0.05 was considered statistically significant.

## Result

### Demographic characteristics

A total of 20 patients with COSTD underwent sequential treatment and implanted 3D-printed protheses at our center from February 2020 to February 2023 meet the inclusion criteria, of which 7 patients had NPWT covering flap closed-incisions after 3D-printed prostheses implantation (NPWT group) and 13 patients did not (Control group). There were no significant differences between the two groups in terms of patient baseline information such as age, gender, ASA classification, and preoperative laboratory indices (including haemoglobin, alanine transaminase, aspartate transaminase, creatinine, prothrombin time, activated partial thromboplastin time, and D-dimer) (Table 2).

**Table 2:** Basic information, demographic characteristics and laboratory indices of patients

		Control Group, *n* = 13	NPWT Group, *n* = 7	*p* value
Age, year		53.4±9.1	52.3±8.9	0.799
Gender, n (%)				>0.99
Male		9(69.2)	5(71.4)	
Female		4(30.8)	2(28.6)	
ASA grading				0.336
I		4(30.8)	0	
II		7(53.8)	6(85.7)	
III		2(15.4)	1(14.3)	
Operation time, minute		269.2±40.5	283.4±30.4	0.427
Bleeding, mL		377.7±257.3	314.3±156.4	0.562
Pre-operation	HGB, g/L	142.9±16.4	140.4±8.1	0.712
	ALT	22.4±5.7	25.9±7.8	0.267
	AST	23.7±7.6	21.9±3.4	0.554
	Creatinine, μmol/L	65.4±10.3	64.4±8.2	0.835
	PT, s	11.9±0.6	11.8±1.2	0.812
	APTT, s	32.3±2.8	32.0±2.1	0.782
	D-dimer, μg/mL	0.19±0.02	0.22±0.08	0.316
Hospitalization time, day		31.6±19.7	19.4±1.7	0.047*
Hospitalization expenses, yuan		144294.8±12108.2	125934.7±9546.8	0.002*

*Abbreviations*: *NPWT* Negative pressure wound therapy, *ASA* American Society of Anesthesiologists, *HGB* Haemoglobin, *ALT* Alanine aminotransferase, *AST* Aspartate aminotransferase, *PT* Prothrombin time, *APTT* Activated partial thromboplastin time

### Surgery and complications

The mean operative time was 283.4 ± 30.4 min in the NPWT group and 269.2 ± 40.5 min in the Control group, with no statistical difference in overall operative time and intraoperative bleeding. During the 14-day postoperative observation period, there was no significant difference in the flap blood supply between the two groups (*p* = 0.051), in which 3 (23.1%) cases of swelling, 2 (15.4%) cases of abnormal skin edge color, and 1 (7.7%) case of low skin temperature occurred in the Control group. And all the flap conditions improved within two weeks after flap nursing and conventional dressing. Conversely, the NPWT group did not have the above-mentioned situation (Table 3). The average length of stay in the Control group increased by over 10 days compared to the VSD group (*p* = 0.047), and there was a significant increase in hospital costs (*p* = 0.002) (Table 2).

**Table 3:** Postoperative flap manifestations of injured blood supply and wound major complications

	Control Group, *n* = 13	NPWT Group, *n* = 7	*p* value
**Injured blood supply**	6(46.2)	0	0.051
Epidermal blisters	0	0	\
Abnormal color	2(15.4)	0	0.521
Cold temperature	1(7.7)	0	1.000
Swelling	3(23.1)	0	0.521
**Major complications**	8(61.5)	0	0.015*
Exudation	7(53.8)	0	0.044*
Infection	3(23.1)	0	0.521
Non-healing	2(15.4)	0	0.521
Dehiscence	1(7.7)	0	1
**ASEPSIS score**			0.001*
0–10	1(7.7)	4(57.2)	
11–20	3(23.1)	3(42.9)	
21–30	6(46.2)	\	
31–40	2(15.4)	\	
>41	1(7.7)	\	

*Abbreviations*: *NPWT* Negative pressure wound therapy

The two groups exhibited a notable difference of ASEPSIS scores in the Mann–Whitney U test (*p* = 0.001). The median scores were 24 in Control group and 9 in NPWT group respectively. Specifically, within the NPWT group, all patients obtained scores below 20 score, with 57.2% of patients scoring below 10 score. Conversely, within the Control group, a significant majority (69.2%) of patients demonstrated ASEPSIS scores surpassing 20 score (Table 3).

### Efficacy evaluations

There was no statistical difference in ADLS scores, LEFS scores, and VAS scores between the two groups. The postoperative LEFS score, VAS score and ADLS score in the Control group were 49.1 ± 2.5, 5.0 ± 1.7 and 71.3 ± 3.1, respectively, which significantly lagged behind the 52.5 ± 1.3, 6.9 ± 1.1 and 74.4 ± 2.9 scores in the NPWT group, and the changes of scores (ΔLEFS, ΔVAS and ΔADLS) were significantly higher in the NPWT group than the Control group (Table 4).

**Table 4:** Postoperative assessment of the therapeutic outcome by LEFS, VAS and ADL score

	Control Group, *n* = 13	NPWT Group, *n* = 7	*p* value
Preoperative LEFS	51.4±1.9	52.1±1.6	0.379
Postoperative LEFS	49.1±2.5	52.6±1.3	0.003*
ΔLEFS score	-2.3±2.2	0.43±0.98	0.006*
Preoperative VAS	5.5±0.9	5.3±0.8	0.528
Postoperative VAS	5.0±1.7	6.9±1.1	0.017*
ΔVAS score	-0.54±1.8	1.6±0.53	0.001*
Preoperative ADLS	69.7±2.6	70.3±2.7	0.638
Postoperative ADLS	71.3±3.1	74.4±2.9	0.043*
ΔADLS	1.2±1.8	4.6±2.4	0.002*

*Abbreviations*: *NPWT* Negative pressure wound therapy, *LEFS* Lower extremity functional scale, *VAS* Visual analogue scale, *ADL* Activity of daily living scale

### Typical cases

**Fig. 2:**
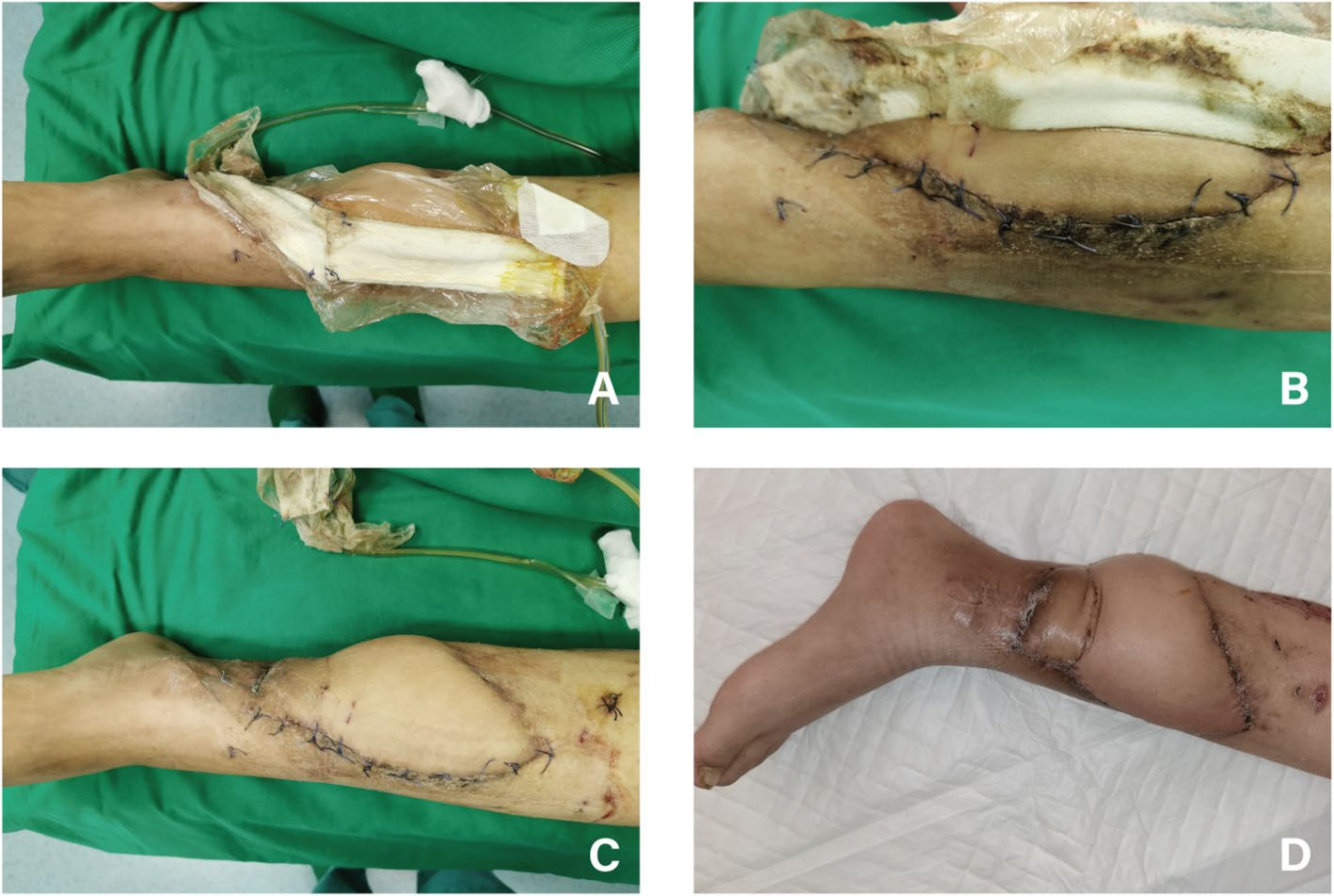
A 58-year-old male patient in NPWT group suffered from COSTD of the left tibia after a car accident and received the sequential treatment. **A** VSD device was used to cover the flap closed-incision after implantation of a 3D-printed prosthesis. **B**-**C** After 2 weeks of negative pressure suction, the VSD device was removed, and a dry and clean closed-incision could be seen. **D** One month after the operation, the flap incision healed well without major complications

**Fig. 3:**
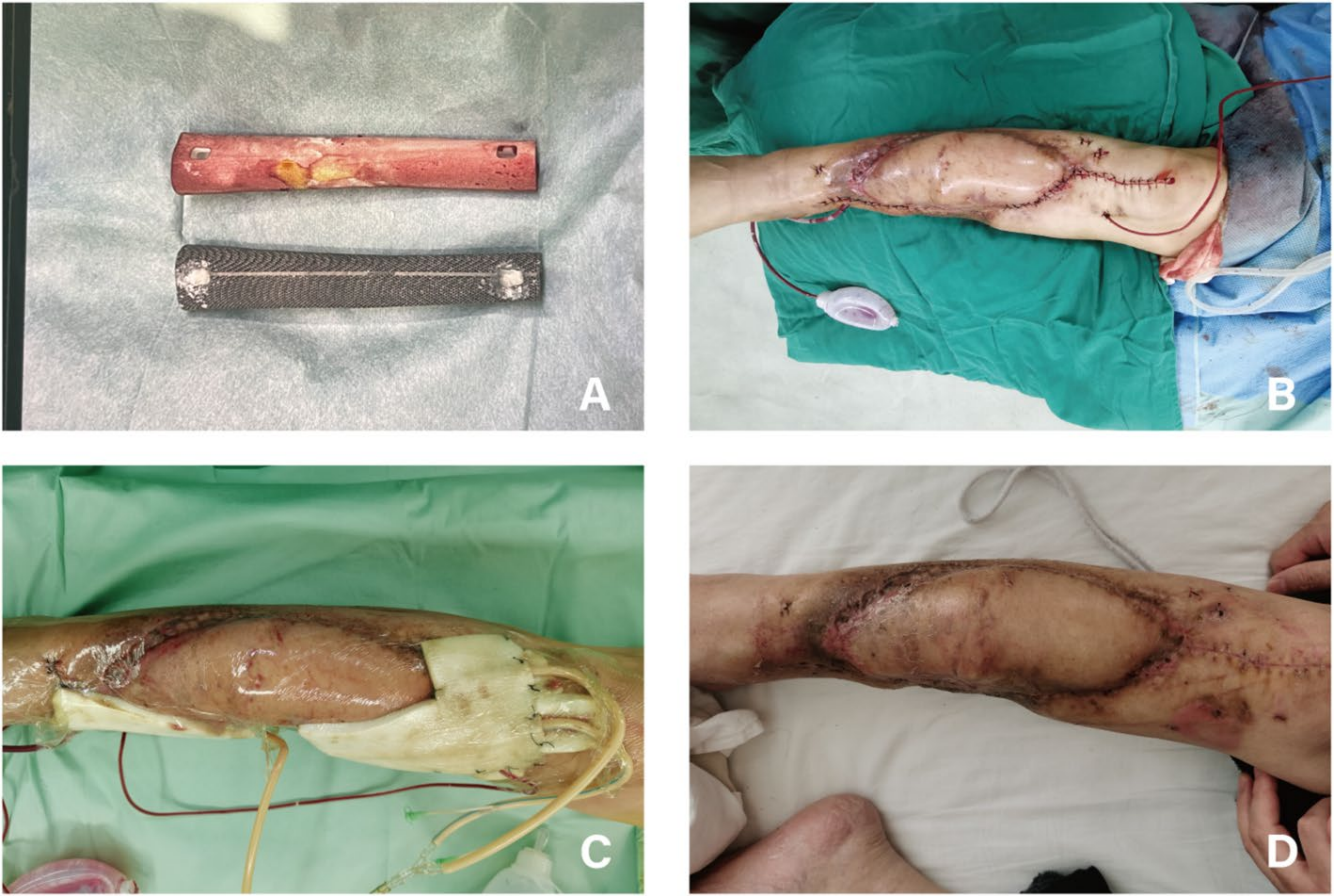
A 62-year-old female patient in NPWT group suffered from COSTD of the right tibia after a car accident and received the sequential treatment. **A** Above was the bone cement Spacer removed during the operation, and below was the 3D-printed prosthesis to be implanted. **B** After the implantation of the prosthesis, the skin incision was intermittently sutured. **C** VSD device was used to cover the flap closed-incision. **D** One month after the surgery, the incision healed well

**Fig. 4:**
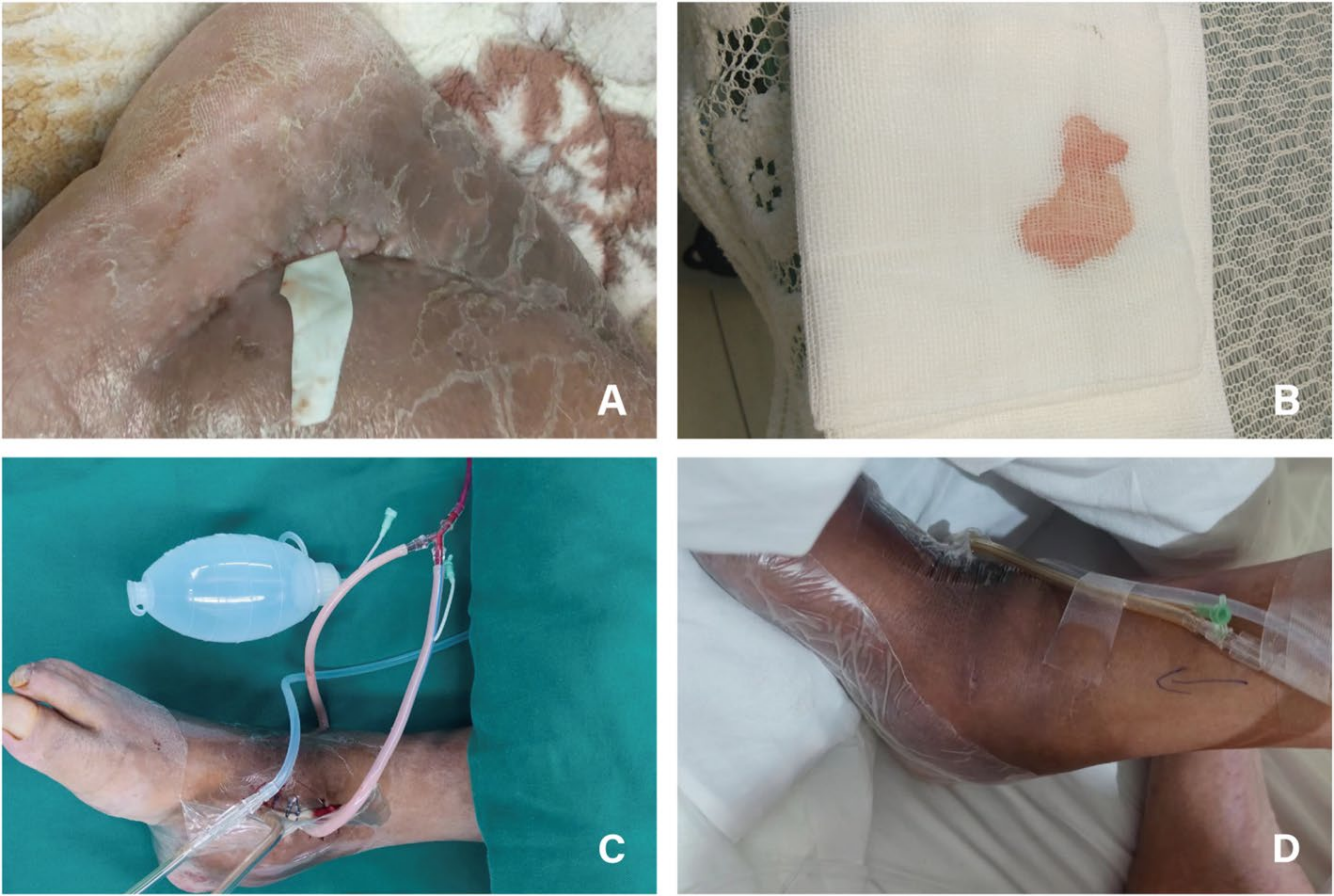
A 65-year-old male patient in Control group suffered from COSTD of the lower right tibia after a car accident, and received sequential treatment in our center. **A** A month and a half after the implantation of the 3D-printed prosthesis, the flap closed-incision showed rupture and exudation, and was treated with rubber band drainage in the outpatient clinic. **B** The exudate was a clear, light red fluid. **C**-**D** The outpatient treatment effect was not satisfactory, and local negative pressure suction was applied with a VSD device during surgery

**Fig. 5:**
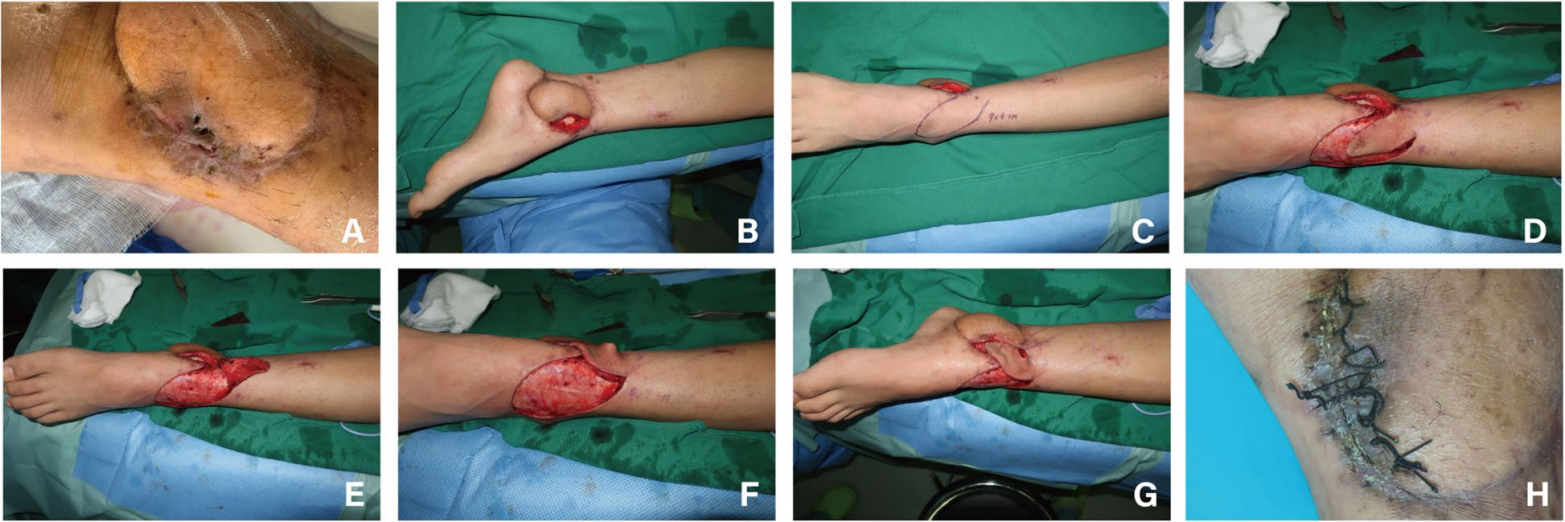
A 56-year-old male patient in Control group suffered from COSTD of the lower left tibia after a car accident, and received sequential treatment. **A** Two months after the implantation of the 3D-printed prosthesis, the flap closed-incision showed rupture and exudation. **B** Thoroughly cleaned the rupture area until the wound surface was clean. **C**-**G** Harvested a local rotational skin flap to cover the wound, and intermittently sutured the incision. **H** Two weeks later, the flap incision healed well

**Fig. 6:**
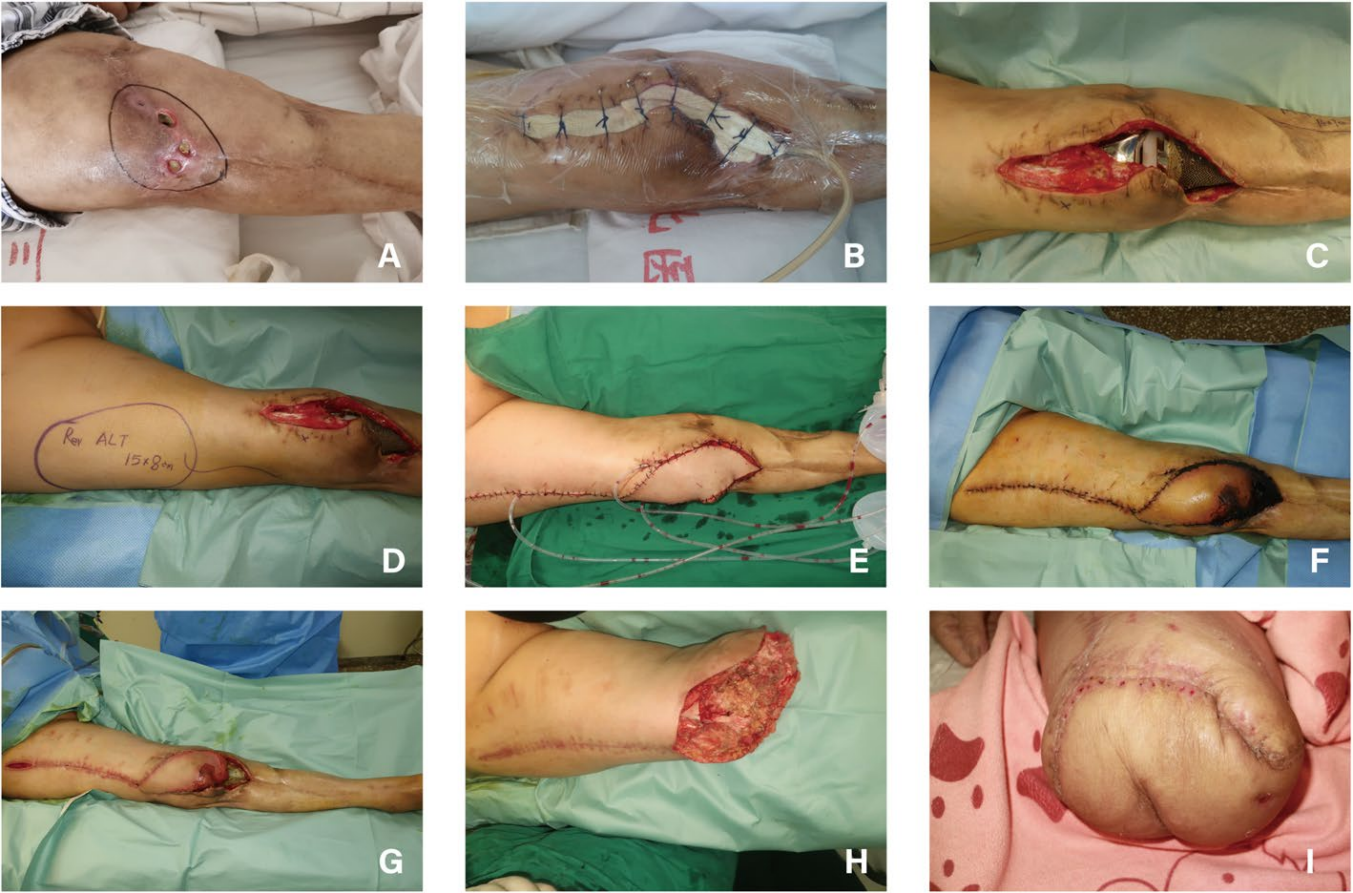
A 71-year-old female in the Control group with chronic osteomyelitis of the right lower extremity (tibia) suffered soft tissue rupture and infection 1 month after 3D-printed prosthesis implantation and received amputation finally. **A** The edge of the flap showed multiple infection and soft tissue ruptures. **B** VSD was placed after debridement. **C**-**E** After the infection being controlled, the reversed anterolateral thigh flap was harvested for the reconstruction of the clean wound. **E**–**G** Necrosis of the distal end of the reversed anterolateral thigh flap and exposure of the prosthesis occurred. **H**-**I** Amputation was performed at a level of 10 cm above the knee

Photographs of five patients were presented. Both Case 1 and Case 2 were patients in the NPWT group. Following prosthesis implantation, VSD devices was utilized to cover the flap closed-incision. The patients’ wounds healed well postoperatively, without major complications observed during the follow-up period (Figs. 2 and 3). Cases 3, 4, and 5 were all patients in the Control group. After prosthesis implantation, conventional dressing changes were employed without the use of VSD devices. In Case 3, the flap closed-incision exhibited rupture and exudation of clear, light red fluid one and a half months following the implantation of the 3D-printed prosthesis. Subsequently, local negative pressure suction was administered using a VSD device during secondary surgery (Fig. 4). In Case 4, the flap-closed incision displayed rupture and exudation two months post-implantation. Comprehensive debridement was conducted in the rupture area until the wound surface was clean, and a local rotational skin flap was harvested to cover the wound (Fig. 5). In Case 5, a 71-year-old female in the Control group with chronic osteomyelitis of the right lower extremity (tibia) experienced soft tissue rupture and infection one month after 3D-printed prosthesis implantation. The edge of the flap exhibited multiple infections and soft tissue ruptures. VSD was applied following debridement. Once the infection was controlled, a reversed anterolateral thigh flap was harvested for reconstruction of the clean wound. However, necrosis of the distal end of the reversed anterolateral thigh flap and exposure of the prosthesis occurred. Ultimately, amputation was performed at a level 10 cm above the knee due to the patient’s poor tolerance and financial burden (Fig. 6).

## Discussion

This study retrospectively studied the details of 20 patients with COSTD who underwent flap margin incision prosthesis implantation at our center, including 7 patients who used NPWT to cover the closed-incisions of flaps postoperatively and 13 patients who didn’t. Our findings revealed that the use of NPWT was effective in reducing the occurrence of postoperative flap wound complications, improving subjective satisfaction, accelerating functional recovery of the lower extremity as well as reducing the corresponding treatment expenditures.

Chronic osteomyelitis is a group of inflammatory diseases involving bone and its surrounding tissues, mostly post-traumatic chronic osteomyelitis in young and middle-aged men [9]. Chronic osteomyelitis occurs in the lower extremities, especially in the tibia, which is the most common site of open fractures [10, 11]. Our team together with our orthopedic department has established a sequential treatment process for COSTD in previous study: (1) systematic antibiotic control of infection; (2) thorough debridement of the infected foci; (3) implantation of bone cement Spacer for occupancy to prevent contracture of the surrounding soft tissues; (4) repeatedly debride the infected site and cover the wound with NPWT during the period until three consecutive negative bacterial cultures; (5) design an appropriate flap for soft tissue wound repair; and (6) perform bone cement Spacer removal and 3D-printed prosthesis implantation after the flap survives well for at least six months [5, 6, 12].

It is evident that the healing of the flap following the implantation of 3D-printed prostheses is particularly crucial [13]. Firstly, if the flap heals poorly or becomes infected, it can result in the prostheses not being covered. This can greatly affect the patients’ limb function and may necessitate further surgery or even amputation [14, 15]. For instance, one postoperative wound infection patient included in this study underwent wound debridement and, after considering her own tolerance and economic burden, ultimately chose amputation. Secondly, both doctors and patients invest significant time and economic resources in the early stages to reach the final stage of this sequential treatment. If it fails at this point, it not only imposes a heavy burden on the patient and their family but also represents a significant loss to medical resources. Furthermore, long-term treatment can easily lead to psychological problems of patients. The discrepancy between the actual treatment situation and the expected efficacy may cause a serious psychological burden for patients and endanger their mental state [16, 17]. Therefore, our center is consistently dedicated to improving the healing of flap closed-incisions following 3D-printed prostheses implantation in clinical practice, creating opportunities for patients to recover their limb function in the future.

Under normal circumstances, flaps are used as tissue to cover wounds and rarely require a second surgery to be cut up, except for flap thinning. However, for patients receiving COSTD sequential treatment, it is unavoidable to incise the flap a second time due to the need to remove the bone cement Spacer and implant the 3D prosthesis [5]. In addition, the flap incision for COSTD differs from that for flap thinning [18, 19]. Firstly, the degree of damage to the flap is different. Unlike conventional flap transfer where the bottom of the flap adheres tightly to the wound tissue, the bone cement Spacer fills the space under the COSTD flap and cannot fully adhere to the flap due to its non-biological properties. The contact interface between the two is weak and loose, and the part of the flap covering the bone cement is easily completely peeled off after incision, causing the flap to be completely lifted. Flap thinning, on the other hand, uses multiple, local, shallow sharp peeling to remove subcutaneous fat. Each opening does not exceed 1/2 of the area of the flap and incisions are minimized to avoid extensive peeling and prevent damage to blood supply to the flap [20, 21]. Secondly, after opening the COSTD flap, it is still difficult for the implanted 3D-printed prosthesis to adhere tightly to the bottom of the flap and rebuild basal blood flow. However, after flap thinning, it is easy for the flap to adhere and heal with basal wound surface and rebuild partial basal blood supply, which helps with flap survival [22]. It can be seen that flap opening in the therapy of COSTD causes more extensive and deeper damage with a greater potential risk of impaired vascular integrity and a higher probability of postoperative complications. This is consistent with our study results where 8 out of 13 Control group patients (61.5%) experienced varying degrees of postoperative complications after flap opening. Although most patients with complications healed well after dressing changes and wound care, some required secondary debridement or even amputation. Therefore, whether good healing of flap closed-incision can be achieved or not after 3D-printed prostheses implantation in the treatment of COSTD are critical to overall treatment success, which is worthy of close attention.

NPWT modulates the expression of growth factors, tissue deformation at micro and macro levels, blood flow, exudation, and bacterial load through biological and mechanical mechanisms, ultimately altering the local wound environment to promote healing [23, 24]. Prior studies have provided compelling evidence for the positive effects of NPWT in facilitating the closure of surgical incisions and minimizing complications [25–27]. Nonetheless, the application of NPWT in flap closed-incisions following the 3D-printed protheses implantation in the treatment of COSTD remains unexplored.

This study enrolled 7 patients who underwent sequential treatment for COSTD and received NPWT for flap closure. During the follow-up period, none of the patients experienced severe complications or discomfort, and the incidence of major wound complications and ASEPSIS scores in the NPWT group was notably lower than that in the Control group. Furthermore, the NPWT group demonstrated significant improvements in postoperative lower limb function score, activities of daily living score, self-satisfaction, and other outcome measures compared to the Control group. These findings highlight the safety and efficacy of NPWT in the flap closure process in the therapy of COSTD, underscoring its capacity to promote local wound healing and expedite the recovery of limb function, thus improving the overall patient experience.

NPWT is a beneficial treatment option for promoting the healing of skin incisions through multiple mechanisms. The placement of a VSD has been shown to effectively reduce the dead space under the skin and drain the seepage and hematoma from under the skin [28]. In this study, it was found that among the 8 patients in the Control group who had complications of flap closed-incisions after surgery, 7 patients had exudation, and the liquid discharged was clear body fluid. We hypothesize that the seepage under the flap closed-incisions after implantation of 3D-printed prostheses differs from that under a regular closed-incision due to the extensive peeling of the flap during the implantation and the material characteristics of 3D-printed prostheses. These factors can result in a less secure adherence between the skin flap and the prosthesis, leading to increased formation of dead spaces and significant leakage and accumulation between them. Such circumstances have a detrimental impact on the recovery of the skin flap wound. Additionally, the presence of a moisture-laden environment surrounding the wound, due to the infiltration of bodily fluids, constitutes a risk factor for further wound infection [29].

Moreover, NPWT enhances blood flow around the wound, and this increased blood supply accelerates the clearance of metabolic waste generated during the cell healing process, while ensuring the provision of nutrients to promote wound healing [30, 31]. Negative pressure suction also improves the degree of edema in the surrounding tissues of the wound by promoting lymphatic reflux, increases the formation rate of granulation tissue, and avoids contact between the wound and the external environment, thereby maintaining a relatively sterile wound [32, 33]. Additionally, NPWT can reduce the relative displacement of the wound edge and provide a more stable wound healing environment through mechanical stability and appropriate pressure on the wound [34].

At the cellular level, NPWT promotes wound healing through a variety of mechanisms, including the transformation of mechanical forces into biological signals, the regulation of cytokine secretion, and the upregulation of signaling proteins such as vascular endothelial growth factor (VEGF), platelet-derived growth factor (PDGF), and fibroblast growth factor 2 (FGF2) [35]. These factors, in turn, promote key aspects of the wound healing process, including angiogenesis, extracellular matrix remodeling, and granulation tissue formation. In summary, NPWT improves the wound environment through both mechanical and biological means, which can accelerate the healing process. In the context of the sequential treatment of COSTD, NPWT has been found to be a consistent and effective adjunct therapy. Its use is indicated from the initial debridement phase through mid-term skin grafting and ultimately closure of the flap incision after implantation of 3D-printed prostheses. The excellent effects of NPWT on controlling infection, improving adherence, and promoting healing have established its vital role in the sequential treatment of COSTD.

This article presents an innovative study that investigates the role of NPWT in the sequential treatment of COSTD in terms of flap closed-incisions healing after 3D-printed protheses implantation. The study demonstrates that NPWT is superior to conventional dressing changes in reducing postoperative complications, accelerating wound healing and lower limb function recovery, while also lowering hospitalization time and costs. However, it is important to acknowledge some limitations of this study. Firstly, the sequential treatment of COSTD has not been widely promoted, and most local hospitals do not have the capacity to treat it. Therefore, many patients with weak limb-saving intentions underwent amputation surgery at local hospitals, resulting in a small sample size for this study. Secondly, being a national central hospital, some out-of-town patients did not return for regular follow-up visits after surgery, leading to missing follow-up data and the inability to include some patients in the study. Thirdly, the overall material was small, and the data was retrospective and there was little possibility to perform adjustment for confounders. In order to address these limitations, we plan to expand the number of patient cases and continue to refine our research in further stages.

## Conclusion

In the sequential treatment of chronic osteomyelitis of the lower extremity with soft tissue defects, the use of NPWT to cover flap closed-incisions after the implantation of 3D-printed prostheses can effectively reduce the occurrence of related major complications and improve patient satisfaction. This approach promotes the recovery of lower limb function and daily activity, ultimately improving the overall success rate of sequential treatment for chronic osteomyelitis with soft tissue defects.

## Data Availability

The data that support the findings of this study are available from the corresponding author with proper reasons.

## Abbreviations

NPWT: Negative Pressure Wound Therapy
COSTD: Chronic Osteomyelitis with Soft Tissue Defects
VAS: Visual Analogue Scale
ADLS: Activity of Daily Living Scale
LEFS: Lower Extremity Functional Scale
VSD: Vacuum Sealing Drainage
VAC: Vacuum-Assisted Closure
ASA: American Society of Anesthesiologists
HGB: Haemoglobin
ALT: Alanine aminotransferase
AST: Aspartate aminotransferase
PT: Prothrombin time
APTT: Activated partial thromboplastin time
